# Touch-Enabled Reversible Microfluidic Ultradense Chips for Convenient, High-Throughput Electrochemical Assays

**DOI:** 10.1021/acsami.5c08760

**Published:** 2025-07-21

**Authors:** Pedro H. N. da Silva, Paula C. R. Corsato, Christian O. Silva, Gabriel J. C. Pimentel, Bruna M. Hryniewicz, Bruna Bragantin, Rodrigo S. Costa, Flávio M. Shimizu, Iris R. Sousa Ribeiro, Renato S. Lima

**Affiliations:** † Brazilian Nanotechnology National Laboratory,Brazilian Center for Research in Energy and Materials,Campinas, São Paulo 13083-970, Brazil; ‡ Institute of Chemistry, 28132 University of Campinas, Campinas, São Paulo 13083-970, Brazil; § Department of Chemistry, Federal University of São Carlos, São Carlos, São Paulo 13565-905, Brazil; ∥ São Carlos Institute of Chemistry, University of São Paulo, São Carlos, São Paulo 13565-590, Brazil; ⊥ Department of Chemistry, Federal University of Paraíba, João Pessoa, Paraíba 58051-900, Brazil; # Center for Natural and Human Sciences, Federal University of ABC, Santo André, São Paulo 09210-580, Brazil

**Keywords:** microfluidics, microfabrication, array, nanomaterial, cell, phosphate

## Abstract

Here, we present a new approach to reversibly bond microfluidic polydimethylsiloxane (PDMS) channels on low-cost, reproducible, scalable, compact, and ultradense multisensor SU-8-coated chips toward high-throughput electrochemical assays. Based on putting the outlets at the bottom of PDMS, the method only needs manually attaching this substrate on a flat surface, thus offering simplicity, throughput, and reversibility. While a plasma-mediated approach failed to provide leakage-free bonding, the reversibly bonded devices presented a high adhesion strength, withstanding a pressure of at least 5.1 MPa. Because the approach is high-pressure tolerant and reversible, it can deliver both long-term analyses and ease of sampling in-channel material for posterior manipulation/characterization and even sensor regeneration. Importantly, the bonding also delivers long-term shelf life and reusability. Three proof-of-concept applications are presented: (i) the electrodeposition of different nanostructured microelectrodes, followed by their downstream characterization and electrochemical tests, (ii) the long-term proliferation and monitoring of colorectal and breast cancer cells through electrochemical cell adhesion assays, along with the following regeneration of sensors and drug susceptibility testing, and (iii) the electrode fouling-amenable determination of phosphate in synthetic body fluids (urine and saliva) for health assessment purposes. High-throughput assays were provided by the chips from fast analyses in series utilizing a hand-held one-channel potentiostat. For instance, 45 analyses could be completed within ∼135 s. One should also note that the approach is compatible with different materials. Hence, future studies can explore this generalizable dry bonding to produce other microfluidic systems for diverse applications.

## Graphical abstract

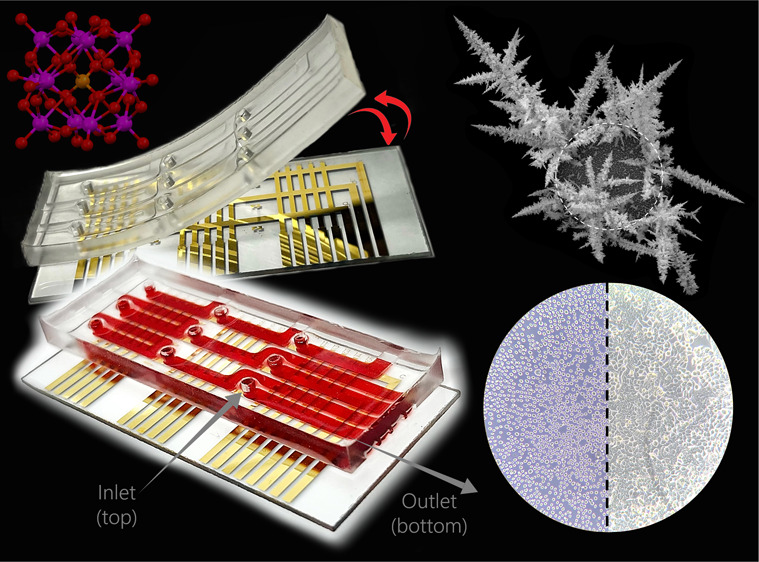

## 1 Introduction

Apart from reducing diagnostic time and ensuring mass testing, high-throughput sensors can boost accuracy and allow the monitoring of coexisting diseases, as they can be used to interrogate diverse biomarkers quickly. While electrochemical single-sensor platforms are an attractive way to enable fast, simple analyses, multisensor devices still face challenges in devising high-throughput tests.^1,2^ These barriers are related to the demand for costly multichannel workstations, cross-talk interferences, and the high number of conductive pads, which limits the engineering of compact ultradense sensing chips to be coupled to microfluidics.^3–10^ To date, microfluidics is a valuable tool by minimizing the consumption of reagents and yielding controlled and dynamic fluid manipulation (μL to pL), hence contributing to boosting throughput and reproducibility further.^11–13^

To advance electrochemical methods toward providing high-throughput and user-friendly tests, our lab recently published a new high-density device referred to as meshed electrochemical chips (MECs).^14–17^ Arranged into a 3D crossbar fashion and vertically separated by SU-8, thin film fingers yield vertical, ultradense two-electrode arrayed sensors (33 to 870 on 75 × 35 mm wafers) while still preserving a low number of conductive pads and lines (each cross point of the fingers forms a sensor). MECs can enhance the throughput by devising fast analyses in series through a practical mode by simply switching the contact of either working (WE) or quasi-reference electrode (QRE) and using a portable, low-cost one-channel potentiostat.

While MECs work well for droplet experiments, the high failure rate of their irreversible bonding with polydimethylsiloxane (PDMS) substrate makes them difficult to implement for microfluidic platforms. Based on exposing the chip and PDMS to N_2_ plasma to introduce amino groups on PDMS (they are expected to allow for bonding with MEC through reactions with residual epoxy groups on SU-8 that is the top chip layer),^18,19^ this bond-ing approach has suffered from a high leakage rate (∼50%), as recently observed in our lab. Here, we present a new approach that allows for reversibly bonding MECs with PDMS substrates without using any active process such as plasma exposure,^11,18,19^ chemical modification of material surfaces,^20–22^ water dehydration,^23^ or use of adhesive tapes^24,25^ as applied for irreversible or reversible bonding. Instead, our approach allows the production of reversible microfluidic devices by just manually putting substrates of PDMS on MEC. Based on putting the fluid outlets at the PDMS bottom to avoid the formation of the ‘water column’ that is produced along outlets through the substrate top, such a straightforward touch-based process enabled leakage-free fluidic operations under different analysis formats, using micropipette and pumps. We refer to these devices as hand-bonded microfluidic chips (HMCs), which presented 45 on-MEC sensors.

By allowing the adaptation of MEC into a microfluidic fashion, the developed reversible bonding boosts the potentialities of this attractive multisensor device toward high-throughput assays. In addition to providing microfluidics-tied merits, as mentioned previously, the addressed method sustains the broad applicability and commercial deployment compatibility of microfabricated ultradense chips as it holds the advantages of an ideal microfluidic device bonding approach, i.e., simplicity, throughput, reversibility, high adhesion strength, long-term shelf life, reusability, and compatibility with a variety of materials, as discussed later. Moreover, the features at first sight antagonistic of high-pressure endurance and reversibility empower the ensuing microfluidic platform to deliver the trade-off between (i) robust, long-term assays and (ii) ease of sampling/collecting in-channel materials for posterior manipulation/characterization^22^ or even regenerating sensors, as demonstrated here.

## 2 Results and Discussion

### 2.1 Reversible Microfluidic Ultradense Chips

PDMS presented nine channels with a rectangular profile in two dimensions, i.e., 3 × 1 mm and 3 mm × 200 μm (Figure S1). As aforesaid, PDMS substrates with fluid outlets on their bottom devised a reversible bonding by being simply placed on SU-8-coated MECs (Figure 1A). The WEs showed circular recessed areas (800 μm in diameter; Figure S2), and each channel embraced five on-chip sensors (Figure 1B,C). It is worth mentioning that MEC combines high resolution and reproducibility with low cost because of the high number of arrayed sensors.^7^

**Figure 1:**
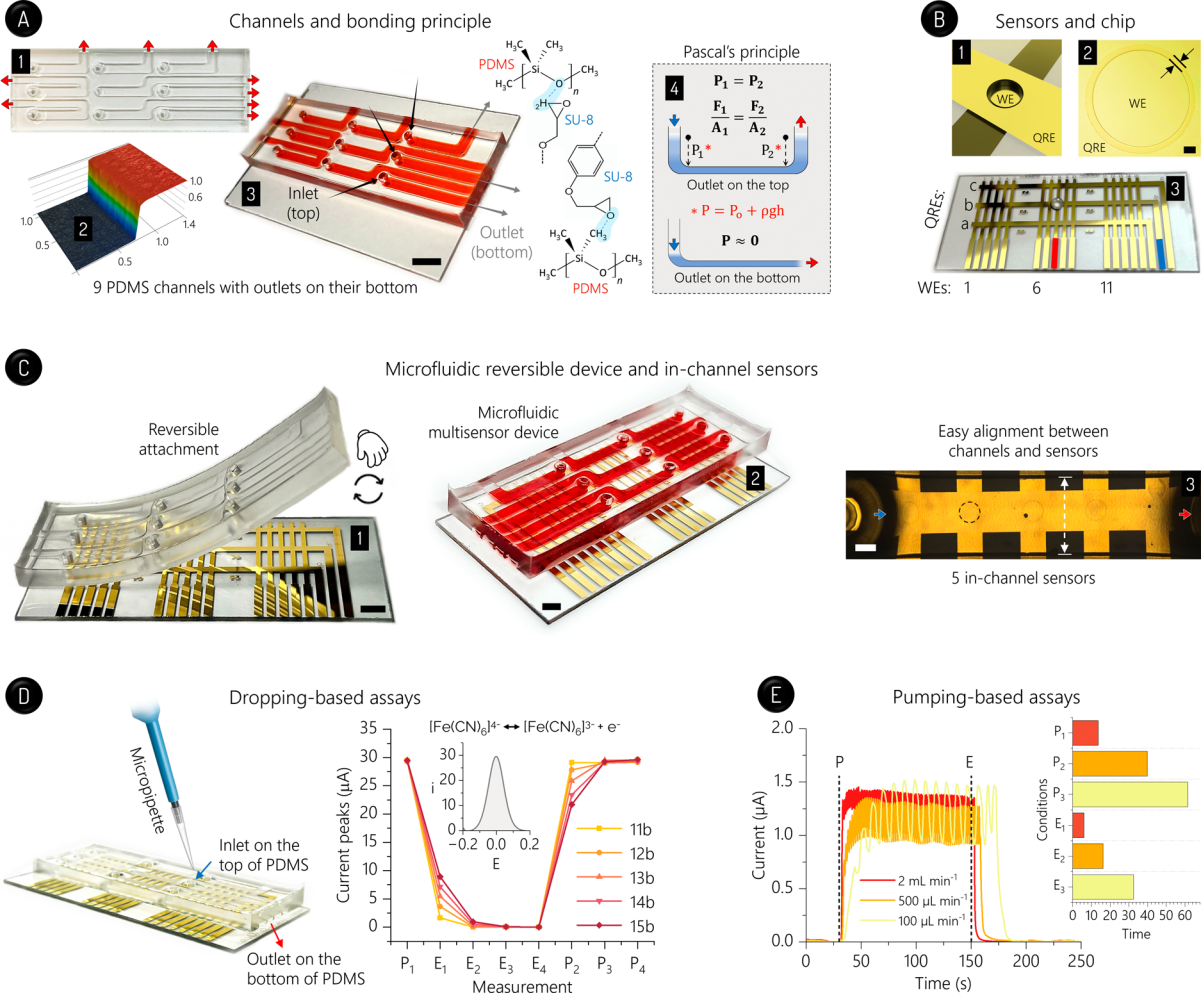
Reversible device and electrochemical analyses in a 3 mm × 1 mm channel to assess the medium exchange. (A) Reversible bonding provided by a PDMS substrate with fluid outlets on its bottom. PDMS with 3 mm × 1 mm channels (1), laser scanning confocal microscopy (LSCM) image (scale in mm) of a channel wall (2), nine channels highlighted by a red dye (3), and Pascal’s principle applied in the cases with outlets placed at the top and bottom of PDMS (4). In (4), *P*, *P*_o_, *F*, and *A* mean hydraulic pressure, atmospheric pressure, force, and area, respectively, whereas ρ, *g*, and *h* express liquid density, acceleration of gravity, and liquid column height, respectively. Insets: illustrations (highlighted in blue) of hydrogen bonds between PDMS and SU-8 monomers (only portions of these monomers are shown). Scale bar: 1 cm (3). (B) MEC with 45 sensors. Illustration (1) and stereoscopy image (2) of a sensor, and MEC with each cross point of the fingers forming a sensor (3). In (2), the SU-8 edges defining the WE area are highlighted by face-to-face arrows. WEs and QREs are identified by numbers and letters, respectively. As an example of the electric contact mode, the colored QRE (blue) and WE (red) in (3) are connected to workstation (sensor: 8b) to analyze the on-chip droplet. Scale bar: 100 μm (2). (C) Reversible device. Chip/PDMS bonding (1), bonded device (2), and inverted light microscopy image of the five in-channel sensors 1a–5a (3). Dashed lines highlight one sensor, and the edges of the PDMS channel are stressed by white arrows in (3). Scale bars: 5 (1,2) and 1 mm (3). (D) SWV peaks (at −0.2 V) recorded by successively dropping 60 μL of electrolyte (E_i_) and 5.0 mmol L^–1^ [Fe­(CN)_6_]^3–/4–^ (probe, P_i_) via a manual micropipette. The sensors 11b and 15b are closest to the inlet and outlet, respectively. Insets: illustration of the assay, usual SWV scan (*i* and *E* mean current in μA and potential in V, respectively), and redox reactions involving the probe. (E) CA currents (at +0.5 V) by alternatively flowing probe (P) and electrolyte (E) using a peristaltic pump. Three flow rates were tested, as indicated in the graphic. Inset: time (in s) needed to reach stable currents for P and E at 2.0 (P_1_ and E_1_), 0.5 (P_2_ and E_2_), and 0.1 mL min^–1^ (P_3_ and E_3_). Blue and red arrows indicate fluid inlets and outlets, respectively, throughout the figure.

Square wave voltammetry (SWV) analyses of 5.0 mmol L^–1^ ferri/ferrocyanide ([Fe­(CN)_6_]^3–/4–^) were first conducted using an all-gold (Au) 45-sensor MEC to evaluate the medium exchange in the PDMS channel via a manual micropipette. Importantly, the electrochemical tests conducted throughout this work were made with a hand-held and user-friendly commercial potentiostat. By serially dropping [Fe­(CN)_6_]^3–/4–^ and electrolyte media (Video S1), the sensors further away from the entrance required a larger volume to reach the control currents for the redox probe and electrolyte. A volume 3 times higher than that of the inner channels covering the five sensors (60 and 180 μL in the two gauged channel dimensions) was needed to ensure efficient medium exchanges for all these sensors, with relative deviations between the reached and control (for channels fully filled with electrolyte or sample, i.e., probe in this test) SWV current peaks being lower than 5% (Figures 1D and S3). In this case, one should underline that the flow originates from the hydrostatic pressure exerted by the droplet volumes filling the channel inlets. The same result was achieved when rinsing solutions directly inside the PDMS channel (Video S2 and Figure S4). The flow in this situation, conversely, stems directly from liquid dispensing and aspiration pipetting steps. To date, the operation of the two-electrode sensors is discussed later in the section 2.5.

HMCs with both channels (3 × 1 mm and 3 mm × 200 μm) also yielded leakage-free continuous operation at flow rates as high as 99 mL min^–1^ (Video S3; specifically, a syringe pump was used to apply this flow rate). In practice, continuous operation of HMCs was further tested employing a peristaltic pump for the chronoamperometry (CA) analysis of 10.0 mmol L^–1^ [Fe­(CN)_6_]^4–^. The ability to tolerate harsh flow rates was advantageous by enabling the platform to reach the control currents faster and reduce the peristaltic pump-yielded pulsing effect on the currents (Figures 1E and S5). In practice, the redox control data (plateau regions) oscillated as relative standard deviations of 16.6, 11.5, and 4.6% at the flow rates of 0.1, 0.5, and 2.0 mL min^–1^, respectively. It is also worthwhile emphasizing that the discard from fluid outlets at the bottom of PDMS was collected on a common channel of a 3D-printed chip holder (Figure S6).

Flow analyses were next sought to evaluate the shelf life and reusability of PDMS to produce HMCs. Substrates stored for as long as 45 days ensured successful reversible bonding and the same PDMS substrate could be reused 5, 10, 30, 60, and 90 days after its preparation. Specifically, these devices could withstand the applied flow rate of 2 mL min^–1^ for 5 min (Figure S7). The same PDMS substrate could further be rebonded against MEC 20 times over two consecutive days, supporting at least 20 mL min^–1^ (1.0 MPa according to burst pressure tests) for 5 min. Besides, while the most irreversible and reversible bonding methods are limited to certain materials,^22,26^ our approach can be easily adapted to other surfaces, e.g., PDMS, glass, and silica (Figure S8). These data support the method’s applicability by confirming its extended shelf life, reusability, and versatility.

### 2.2 Adhesion Strength of Bonding

The adhesion strength of MEC/PDMS devices was quantitatively confirmed through burst pressure tests using 3 mm × 200 μm channels. The touch-based reversible bonding of glass with PDMS bearing outlets from its top side has adhesion strengths ranging from 0.04 up to 0.16 MPa.^27,28^ Here, while a burst pressure of 2.9 MPa was obtained when merging this type of PDMS substrate with SU-8-coated MEC after exposition to N_2_ plasma for 5 min, the HMCs with PDMS carrying outlets from its bottom could meaningfully withstand a pressure of at least 5.1 MPa (i.e., a flow rate of 99 mL min^–1^; Figure S9). This enhancement in pressure endurance makes the device more robust and feasible for long-term assays and applications requiring pressurized pumping and control.^22,29,30^ Here, among the two assessed platforms, only the devices reversibly bonded against PDMS with on-bottom outlets could enable leakage-free 24-h proliferations (at 37 °C) and subsequent electrochemical monitoring of cancer 2D cells, as will be discussed later. In opposite, the devices that were irreversibly bonded against PDMS bearing on-top outlets incurred leakages over device handling or micropipette-assisted microfluidic operations.

The hypothesis proposed here for the adhesion of HMCs is supported by the data when trying to irreversibly attach MEC to PDMS carrying both inlets and outlets from its top side (after 5 min exposition to N_2_ plasma). In this case, more specifically, leakages usually took place during the filling of the waste outlet channel, thus revealing that the enhanced adhesion strength of our touch-based reversible bonding is likely to come from the placement of fluid outlets at the PDMS bottom. First, as verified in any dry adhesive or adhesion-based bonding, one should emphasize that the addressed bonding relies on van der Waals intermolecular forces. Here, hydrogen bonds and hydrophobic interactions are expected to occur under a distance range of 1 nm^26^ between oxygen-with PDMS and SU-8 (polymer that defines WE areas, coating the chip top) surfaces. Moreover, the strength of this adhesion is expected to be boosted when putting outlets at the bottom of PDMS, as this fashion prevents the hydrostatic pressure-caused downward forces from acting in channels (see Figure 1A). This pressure (produced by the fluid column height) was found to be 0.1 MPa in the device with on-top fluid outlets that was tested here (Supporting Information).

### 2.3 Nanostructured Microelectrodes

We next delved into challenging the HMCs with a first analytical proof-of-concept case, i.e., the electrodeposition, characterization, and application of nanostructured microelectrodes (NMEs). These electrodes have drawn interest in the sensor community for their advantages in providing both antibiofouling ability and sensitivity, thus enabling accurate liquid biopsy in bodily fluids.^31–38^ In particular, electrodeposition on recessed WEs (as noted in MEC) is an attractive method as it is compatible with mass-producible thin-film electrodes and faster than the growth on larger planar surfaces. Furthermore, this process makes it possible to attain NMEs spanning from smooth spheres to finely nanorough spiky or flaky structures by altering experimental conditions, hence allowing for the modulation of sensitivity and dynamic range.^39,40^ This feature is advantageous since the clinically important concentrations of biomarkers can vary over many orders of magnitude in patients’ samples.^41^

Electrodepositions in the presence of 25.0 mmol L^–1^ gold­(III) chloride ([AuCl_4_]^−^) were made on HMCs with WEs presenting 45-μm diameter under moderate- (−0.2, −0.3, −0.5, and −0.6 V vs Au) and fast-growth regimes (−1.0 V vs Au) until the charge reached 120 μC.^15,39^ Compared with the droplet-based growth,^15^ using reversible microfluidic on-chip sensors boosted simplicity and throughput. To date, the solutions were added to channels via a manual micropipette, whereas the electrodepositions were performed under a stationary condition. As a further merit coming from the possibility to detach and rebond PDMS on MEC, the HMC allowed the posterior steps of SEM imaging of the on-chip NMEs and SWV analyses of [Fe­(CN)_6_]^3–/4–^ to scrutinize their sensitivity (Figure 2A).

**Figure 2:**
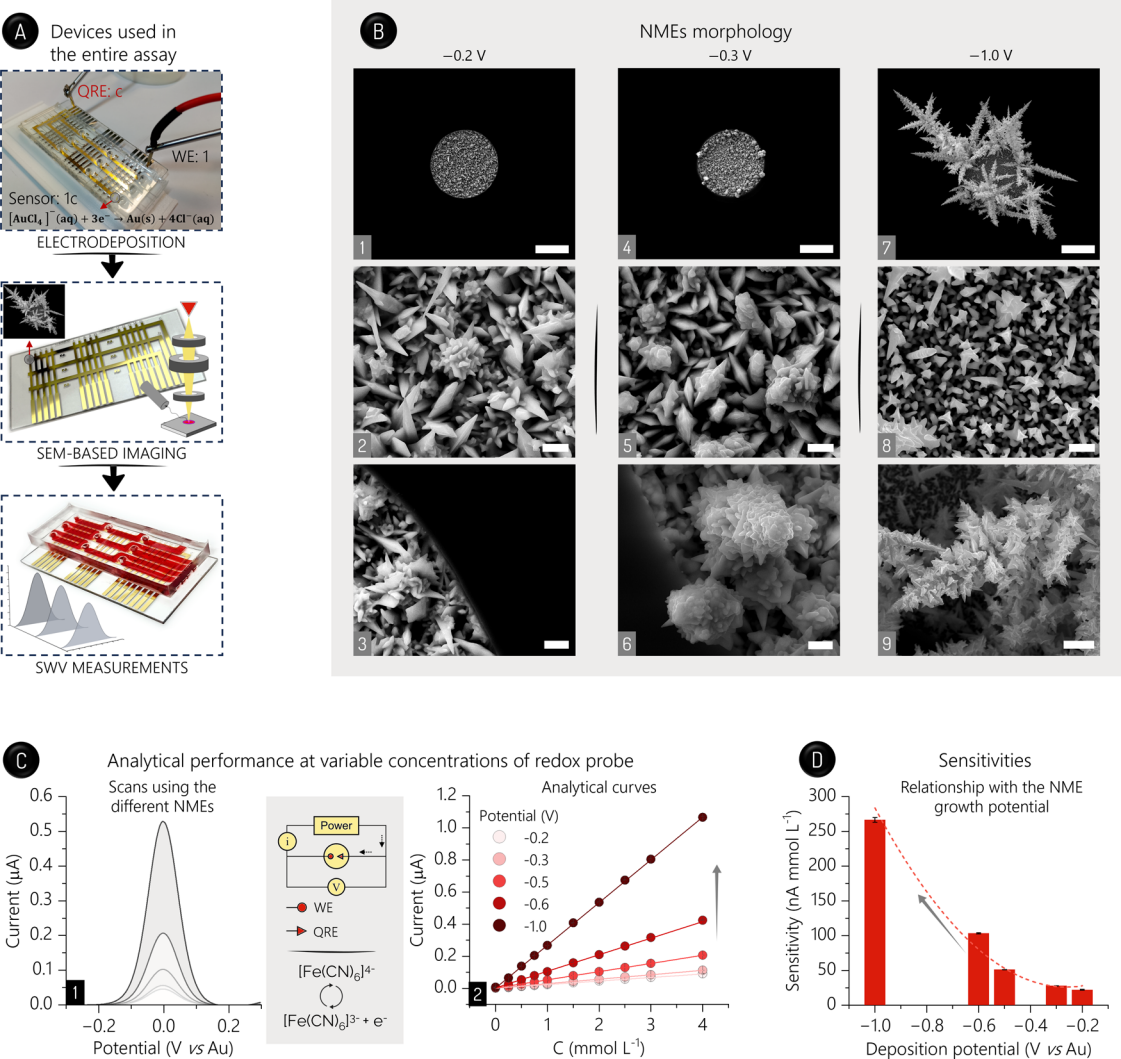
NMEs. (A) Devices used along the assays, namely, the bonded device for NME growth, the chip for SEM imaging after PDMS detachment, and again the bonded device for SWV analyses after rebonding with the same PDMS piece. In the top picture, the electrodeposition reaction taking place on the activated sensor (1c in this image, as highlighted) is shown. (B) SEM images. NMEs obtained by applying −0.2 (1–3), −0.3 (4–6), and −1.0 V (7–9). Scale bars: 20 μm (1, 4, and 7), 1 μm (2, 5, and 8; middle of WEs), and 2 μm (3, 6, and 9; edges of SU-8 hole). (C) Analytical performance. SWV scans for the probe (2.0 mmol L^–1^) using NMEs obtained under −0.2, −0.3, −0.5, −0.6, and −1.0 V (bottom to top signals; 1) and analytical curves (*n* = 5) using the NMEs obtained at distinct potentials, as stressed (2). Insets: generic circuit of the two-electrode on-MEC cells and reactions involving the probe. (D) Relationship between sensitivity and electrodeposition potential. Solutions were added to channels using a manual micropipette.

According to scanning electron microscopy (SEM) images, variations in the applied potential meaningfully influenced the morphology of NMEs (Figures 2B and S10), as expected.^39^ Electrodeposition at −0.2 V vs Au was confined within the SU-8 hole, with the NMEs consisting of leaf- and, especially, spike-like structures. At potentials ranging from −0.3 to −0.6 V, conversely, leaf-like structures reaching the top of SU-8 also began to be produced at the WE edges, gradually becoming larger and more abundant as the potential increased. When the cathode potential increased to −1.0 V, these leaf-like NMEs were specially generated at the WE edges, rendering more nanostructured textures. These morphologies can be explained by nucleation and growth theory, as discussed next.

At low potentials, there is the progressive nucleation of on-surface (primary) nuclei co-occurring with growth. These primary nuclei are gradually and slowly formed over time under a constant replenishment rate of [AuCl_4_]^−^ ions, thus triggering the isotropic formation of smooth, bulky spherical structures via the reduction of Au^3+^(aq) to Au^0^(s).^39^ Continuing, both isotropic and anisotropic growths are verified at intermediate potentials, incurring rough and heterogeneous textures. In addition to the isotropic progressive nucleation of primary nuclei, a second but anisotropic instantaneous nucleation emerges across the sensor surface. During this process, the so-called secondary nuclei (on top of the primary nuclei) are created instantaneously, followed by the growth step. Since this instantaneous nucleation is faster and involves a number of active sites smaller than the progressive nucleation, the Au growth occurs heterogeneously, implying the formation of anisotropic NMEs. Finally, the high-potential electrodepositions are driven by an anisotropic process because both secondary and primary nucleations undergo instantaneous regimes. In this case, rapid Au^3+^/Au^0^ redox reactions yield the growth of highly rough, finely nanostructured NMEs.

From the prior discussion, the hybrid isotropic/anisotropic regime is supposed to drive growth at the potentials from −0.2 to −0.6 V. The larger the negative potential, the rougher and more structured the NMEs were, thus indicating that the anisotropic contribution increased with the potential. Conversely, a highly anisotropic regime was probably reached at −1.0 V, when finely nanostructured NMEs were extensively produced around the edges of SU-8 hole (see Figures 2B and S10). To date, the predominant growth of these on-edge Au structures can be ascribed to the highest potential that is established in this region under certain fast-growth conditions.^39^

In contrast to the nanoporous structures that are commonly electrodeposited from recessed microelectrodes (up to 10 μm in diameter),^33,34,40^ nanopore-free spiky structures are achieved on MEC, rendering the resulting sensor capable of increasing the sensitivity of diffusion-limited assays.^15^ This property was con-firmed here via SWV analyses of the [Fe­(CN)_6_]^3–/4–^ at different concentrations (*n* = 4). The sensitivity increased exponentially with the potential (Figures 2C,D, and S11), which is probably owing to the generation of more nanostructured Au NMEs (i.e., with higher electroactive area) as the potential increases.

### 2.4 Cell Proliferation and Drug Susceptibility Tests

Having interrogated HMCs with the electrodeposition, characterization, and sensing performance evaluation of nanopore-free NMEs, the device was next scrutinized as a multifunctional platform for the long-term on-WE proliferation and monitoring of colorectal (HT-29 line) and breast (MDA-MB-231 line) 2D cancer cells (Figure 3A), along with the regeneration of sensors. These analyses relied on cell adhesion assays by detecting 6.0 mmol L^–1^ hexaammineruthenium­(II) ([Ru­(NH_3_)_6_]^3+^) with SWV using 800-μm WEs. By gauging cellular proliferation or adhesion, the method could be used to foresee tumor formation, cancer cell aggressiveness, and wound healing ability (regenerative medicine), along with screening the efficacy and cytotoxicity of anticancer drugs and recognizing immune systems.^42^

**Figure 3:**
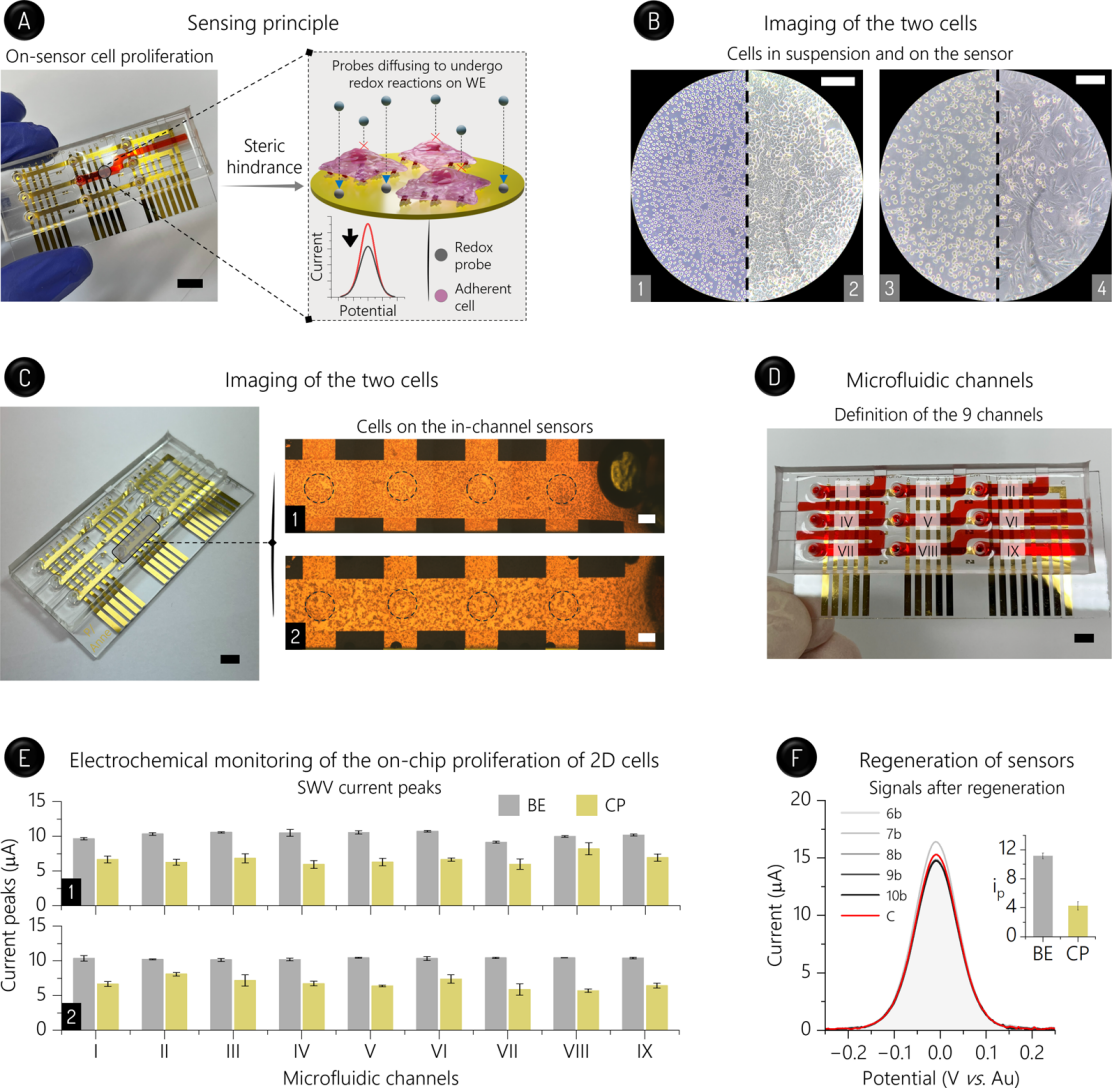
Cell proliferation and sensor regeneration. (A) Detection principle based on the cell-induced steric hindrance against the probe undergoing on-WE redox reactions. Scale bar: 5 mm. (B) Optical images of HT-29 (1,2) and MDA-MD-231 (3,4) 2D cells in suspension (1,3) and adhered on WE after 24-h proliferation (2,4). Scale bars: 20 μm. (C) Inverted light microscopy images of the HT-29 (1) and MDA-MD-231 (2) cells grew along the PDMS channel. Black dashed lines highlight the four sensors (of a total of five) that could be seen in this image. Scale bars: 1 cm. (D) Identification of the nine PDMS channels (I–IX) that was adopted in this article. Scale bar: 5 mm. (E) Cell adhesion SWV assays. SWV peaks (*n* = 15) for 6.0 mmol L^–1^ [Ru­(NH_3_)_6_]^3+^ before (BE) and after proliferation (CP) of HT-29 (1) and MDA-MB-231 cells (2). These data are related to the five sensors enclosed in the channels (I–IX). (F) Regeneration of the sensors 6b–10b after proliferating the HT-29 2D cells. SWV scans for 2 mmol L^–1^ [Fe­(CN)_6_]^3–/4–^ using the regenerated and control (C; i.e., bare) sensors. Inset: global SWV peaks (*n* = 15) related to HT-29 cell adhesion tests using regenerated sensors. Media were added to channels using an automatic micropipette.

After culturing the 2D cells, the resulting medium was introduced into the nine PDMS channels with the aid of an automatic micropipette, followed by in vitro cellular proliferation in a CO_2_ incubator at 37 °C for 24 h and the subsequent addition of [Ru­(NH_3_)_6_]^3+^ for SWV tests. While leakages were noted when using both microfluidic devices, bonded after N_2_ plasma exposure and reversibly bonded with PDMS bearing on-top outlets, their high-adhesion property enabled the HMCs to yield 24-h proliferations and subsequent SWV assays. The absence of hydraulic pressure acting on channels (see Figure 1A) is supposed to make the device viable for long-term analyses, as aforesaid.

The cells were dropped in the nine PDMS channels under the concentrations (10 × 10^4^ and 15 × 10^4^ cells for MDA-MB-231 and HT-29 lines, respectively, per PDMS channel) that implied high-density and homogeneous proliferation on five in-channel sensors (see Figure 3B,C). These data also confirm the biocompatibility of sensors. Because each SWV scan lasted 3 s, one should also stress that the electrochemical analyses using the 45 on-MEC sensors (measurements in triplicate) could be finished within only ∼405 s.

The SWV current peaks for [Ru­(NH_3_)_6_]^3+^ were reduced by 35 ± 7% (HT-29 line) and 35 ± 8% (MDA-MB-231 line) due to the steric hindrance yielded by adherent proteins, which are responsible for the cellular adhesion (see Figure 3A).^17^ Importantly, the data for both lines were reproducible (Figures 3D,E, and S12), indicating both that the pipetting steps did not lead to cell detachments and that potential interferences, e.g., the cell adhesion taking place on all sensor elements (QRE, SU-8, and WE; see Figure 3C) and electrode fouling caused by biomolecules present in the cellular medium, had no effective impact on the good working of sensors, which agrees with our prior results.^17^ While electrode fouling, e.g., is expected to occur over cell proliferation, this phenomenon is presumed to be systematic across all sensors, implying no damage to the capability of sensors to monitor the on-WE cellular attachment/detachment processes.

In addition to devising user-friendly, high-throughput, and reproducible electrochemical tests of cell proliferation with the aid of an automatic micropipette, the HMCs allowed for the regeneration of the sensors. Briefly, the PDMS substrate was manually removed from the top of the MEC after cell assays, and the sensors were then dipped into a sodium hypochlorite solution to obtain cell-free surfaces. Next, the chip was exposed to N_2_ plasma to reactivate the electrodes and reversibly rebonded. The regenerated sensors could recover their original performance, as confirmed through SWV analyses of 2 mmol L^–1^ [Fe­(CN)_6_]^3–/4–^ and new cell adhesion assays for HT-29 cells (Figure 3F). In the latter case, the SWV peaks for 6 mmol L^–1^ [Ru­(NH_3_)_6_]^3+^ decreased by 38 ± 5%.

As previously mentioned, the method can be used for screening the efficacy of anticancer drugs by assessing cellular adhesion. This application was preliminarily demonstrated here after exposing the proliferated HT-29 cells (15 × 10^4^ cells per PDMS channel, as adopted before) to four concentrations (5, 50, 100, and 200 μmol L^–1^) of doxorubicin (DOX), an anticancer drug. These so-called drug susceptibility tests relied on cell detachment electrochemical assays,^17^ in which the SWV peaks for 6.0 mmol L^–1^ Ru­(NH_3_)_6_^3+^ (added to solution) were directly proportional to the concentration of DOX (Figure S13), as expected, because the cells detached from the WE surfaces as they died. Importantly, the drug effect was assessed through the parameter termed cell index (CI) that ranges from 0 to 1, with these values meaning 100% drug-triggered cell death and no drug effect, respectively. The CIs were calculated as^17^

$$\mathrm{CI}=\frac{I_{p}^{\mathrm{DE}}-I_{p}^{\mathrm{No}\mathrm{cell}}}{I_{p}^{\mathrm{CP}}-I_{p}^{\mathrm{No}\mathrm{cell}}}$$ 1

wherein *I*_p_^Nocell^, *I*_p_^CP^, and *I*_p_^DE^ mean the SWV current peaks collected for Ru­(NH_3_)_6_^3+^ before cell proliferation, just after proliferation, and after DOX exposure, respectively.

In practice, solutions with the four DOX contents were added to the distinct PDMS channels, with the probe, i.e., 6 mmol L^–1^ [Ru­(NH_3_)_6_]^3+^, being next serially interrogated with SWV using the five in-channel sensors. Because each SWV scan lasted 3 s, as aforesaid, the analyses of the four samples (a total of 20 sensors; measurements in triplicate) was finished within only ∼180 s. Remarkably, the CIs at increasing DOX concentrations were in line with the cell viabilities, the parameter that is conventionally used in susceptibility tests to evaluate drug effects (Figure S14). These data suggest a promising potentiality of the method to enable high-throughput drug screening. Critically, the lack of platforms for providing these analyses over the preclinical trials of drug candidates remains a barrier to rapidly developing safe and effective medicines.^17^

### 2.5 Phosphate Analysis

To emphasize the significance of high-throughput analytical assays, the third analytical proof-of-concept case consisted of determining phosphate in synthetic bodily fluids by being a relevant biomarker for clinical diagnoses. Phosphorus, predominantly present as phosphate ions, is the second most abundant mineral in the human body and is critical for skeletal integrity, energy metabolism, and cellular functions.^43^ Dysregulation of phosphate homeostasis is associated with crucial clinical consequences. Hypophosphatemia (physiological phosphate concentrations below 1266 and 317 ppm in urine and saliva, respectively) may result in skeletal deformities (rickets), muscular dysfunction, respiratory failure, and increased mortality, particularly in critically ill and septic patients.^44,45^ Conversely, hyperphosphatemia (physiological phosphate concentrations above 3165 and 886 ppm in urine and saliva, respectively), frequently linked to chronic kidney disease, is a major contributor to cardiovascular morbidity, atherosclerosis, and decreased survival rates, with phosphate elevations correlating with enlarged mortality risks.^46,47^ Therefore, accurate determination of phosphate in bodily fluids (e.g., urine, and saliva) is crucial for the early diagnosis and management of related pathologies.^48,49^

Phosphate itself is not an electroactive component; nonetheless, a rapid and selective strategy to make its electrochemical determination possible relies on the formation of phosphomolybdate complex through an instantaneous reaction with ammonium molybdate for downstream SWV sensing,^50^ as employed here. A visual confirmation of this complex formation could be observed via a color change from transparent to yellow (Figure 4A). Phosphoric acid was used as the phosphate standard to construct the analytical curves by adding the solutions to channels using again a manual micropipette. In the SWV scans (Figure 4B), the cathodic polarization showed two peaks relative to the reductions of Mo­(VI)–Mo­(IV) and Mo­(IV)–Mo­(II), respectively.^51^ In line with the literature,^50^ the currents from Mo­(IV)–Mo­(II) reduction (at around −0.3 V) were selected as analytical responses of the analyses, which were performed under stationary droplet and microfluidic conditions. It is worth noting that each scan lasted again 3 s, so the interrogation of 9 samples in the microfluidic channels (a total of 45 electrodes) was completed within ∼135 s.

**Figure 4:**
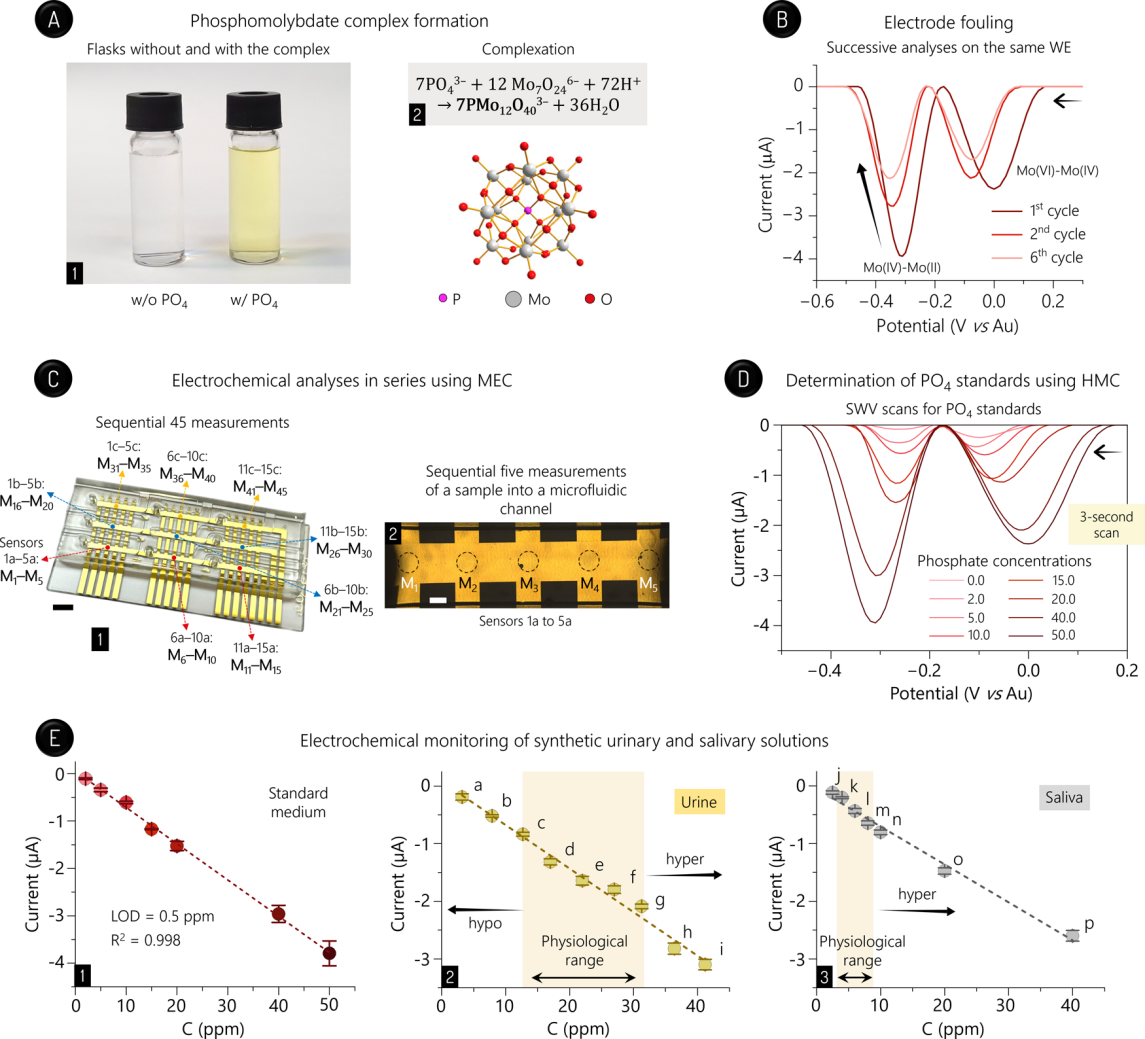
Phosphorus analysis. (A) Complex formation. Visual confirmation of the electroactive phosphomolybdate complex generated after letting phosphorus react with molybdate ammonium (1), together with this complexation reaction and the complex structure (2). (B) Serial SWV scans, as indicated, for phosphate standards at 50.0 ppm. The two peaks relative to the phosphomolybdate complex reduction are shown. (C) SWV analyses in series using the chip. Indication of the 45 measurements (M_1_–M_45_) along the nine PDMS channels (1) and 5 measurements (M_1_–M_5_) utilizing the sensors 1a–5a (2). Scale bars: 5 (1) and 1 mm (2). (D) SWV scans for phosphate standards at distinct concentrations (ppm), as indicated. (E) Analytical performance (*n* = 5). Analytical curve for phosphate standards (1), and curves related to the monitoring of synthetic bodily fluids with standard phosphonate additions, i.e., 320, 790, 1270, 1700, 2200, 2700, 3127, 3640, and 4120 ppm in urine (a–i; 2), and 250, 400, 600, 800, 1000, 2000, and 4000 ppm in saliva (j–p; 3). The physiological ranges for each sample with no incidence of hypophosphatemia (hypo) and hyperphosphatemia (hyper) are indicated in (2,3). Solutions were added to channels employing a manual micropipette.

It should be mentioned that fouling effects were observed due to the adsorption of the phosphomolybdate complex on the WE surface, with the currents pointedly decreasing over successive SWV scans (see Figure 4B).^50,51^ For this reason, a new electrode was used after each measurement, reinforcing the significance of a large number of on-MEC sensors for providing high throughput via fast and serial analyses. For instance, all 45 on-MEC sensors could be interrogated with 3-s SWV analyses in series to monitor nine samples (*n* = 5 for each sample added to a specific PDMS channels, with one measurement per sensor) within ∼135 s (Figure 4C and Video S4).

The peak potentials varied by more than 50.0 mV (−259.3 to −310.7 mV) for distinct concentrations of standard phosphate (Figure 4D; similar results were achieved in the synthetic samples analyzed next). This data can univocally be ascribed to two properties of sensors, i.e., the use of QREs (rather than employing a true reference electrode) and two-electrode cells, with the QRE also taking the function of maintaining the overall system electroneutrality (see Figure 2C). In this way, the sensors are especially prone to current draws through QRE since the potential measurement-related input impedance is not high.^52^ Nonetheless, although the variations in peak potentials, HMCs could yield high-performance phosphate determination, as discussed following.

Compared with the stationary analyses (Figure S15), the microfluidic tests yielded lower standard deviations and a broader linear range in the analytical curve for phosphate standards (Figure 4E). The limit of detection (LOD) and sensitivity were revealed to be 0.5 ppm and −82.2 μA ppm^–1^ for the microfluidic MEC, whereas these same parameters were obtained as 0.9 ppm and −43.8 μA ppm^–1^, respectively, for stationary measurements. We further challenged the HMCs with complex matrices, i.e., synthetic urine and saliva, whose physiological phosphate concentrations range from 1266 to 3165 ppm and 317 to 886 ppm, respectively. Phosphate standards were added to synthetic urine (320, 790, 1270, 1700, 2200, 2700, 3127, 3640, and 4120 ppm) and saliva (250, 400, 600, 800, 1000, 2000, and 4000 ppm) solutions to cover concentrations related to hypophosphatemia, hyperphosphatemia, and normal physiological conditions. To adjust the phosphate concentrations in the bodily fluid solutions within the analytical curve range, all samples were diluted 100-fold. Such contents were determined using the linear regression of the curve achieved for standard solutions. The recoveries ranged from 88 to 106% and 87 to 113% for urine and saliva, respectively (Figure S16), suggesting that our high-throughput system is a promising approach to accurately quantifying clinically relevant phosphate contents in biological matrices.

## 3 Conclusions

In summary, we have developed a reproducible, scalable, low-cost, reversible, high-pressure tolerant, and stable microfluidic multisensor chip toward high-throughput electrochemical tests from multiple, fast analyses in series. While the previously addressed meshed chip holds the merits of reproducibility, mass-production compatibility, cost-effectiveness, and high density of sensors,^14–17^ the reversible dry bonding proposed here offers long-term stability, simplicity, and throughput, as it is simply based on placing the outlets at the PDMS bottom. Importantly, since the approach meets the trade-off between high adhesion strength and reversibility, it can yield both long-term assays and ease of sampling in-channel material for downstream manipulation and even sensor regeneration. These potentialities have been demonstrated across three proof-of-concept applications, as previously presented.

By exploiting the merits of the ultradense chips and reversibly dry bonding, we have created an advanced electrochemical multisensor device toward high-throughput tests, which is a relevant goal in analytical science for further reducing diagnostic time, delivering mass testing, boosting accuracy, and allowing the monitoring of coexisting diseases. While the N_2_ plasma-mediated method failed in bonding SU-8-coated MEC, the HMCs presented a remarkable adhesion strength, yielding leakage-free fluidic operations utilizing either micropipettes or pumps. Other merits of the approach that make it deployable in daily applications are a long-term shelf life and reusability. All these bonding-enabled advantages preserve a broad applicability and commercial development capability of MEC. One should also emphasize that our touch-enabled bonding can save cost and time. Briefly, while plasma-based traditional bonding processes demand exposure times on the order of 5 min, the approach addressed here is practically instantaneous (see Video S4). Moreover, the cost resulting from plasma usage was estimated to be roughly $3.33 per batch (Supporting Information). In a mass-production scenario, these gains in time and cost by HMCs can play a significant role in reducing final costs further over conventional microfluidic devices.

It is also worthwhile mentioning that the addressed reversible bonding is compatible with distinct materials. Therefore, future works can also explore it to produce other microfluidic systems for diverse purposes. For instance, the culturing of in vitro cell models can benefit from the developed touch-based bonding by allowing the sampling of on-chip-grown cells for downstream manipulation and characterization experiments.^22^ Importantly, these models are widely used to understand the drug actions and screen their efficacy/toxicity over the course of preclinical trials by the pharmaceutical industry and personalized oncology.^17^

## 4 Material and Methods

### 4.1 Material

All the reagents were of analytical grade. The solutions were prepared utilizing ultrapure water (Millipore Milli-Q system; 18.2 MΩ cm resistivity at 25 °C). Potassium nitrate (KNO_3_), potassium chloride (KCl), [Fe­(CN)_6_]^3–/4–^, [Ru­(NH_3_)_6_]^3+^, chloroauric acid (HAuCl_4_; Au grow precursor), hydrochloric acid (HCl), dibasic sodium phosphate (Na_2_HPO_4_), monobasic sodium phosphate (NaH_2_PO_4_), DMEM (Vitrocell Embriolife), Trypsin (Vitrocell Embriolife), fetal bovine serum (Gibco Ther-moFisher), penicillin-Streptomycin (Gibco ThermoFisher), PBS (10.0 mmol L^–1^, pH 7.4), and phosphate buffer (PB) were purchased from Sigma-Aldrich (St Louis, MO). PDMS and Sylgard 184 silicone curing agent were supplied by Dow Corning.

### 4.2 Microfabrication

The fabrication of MECs was based on photolithography steps and electron-beam evaporation.^14–17^ Briefly, stacked layers were deposited on glass wafers (75 mm × 35 mm) in the following order: (i) WE fingers, (ii) SU-8, and (iii) QRE fingers. The WE and QRE fingers consisted of 20 nm (in thickness) Cr films (adhesion on-glass layer) underneath 100 nm thick Au films (active upper layer). The detection areas of WEs were photolithographically defined by SU-8 (∼4 μm in thickness), showing circular apertures of 800 μm in diameter. Dimensional data regarding the width (*w*) and length (*l*) values are presented following: *w* = 1.6 mm and *l* = 10.0 mm for the electric contact pads, *w* = 1.0 mm and *l* = 22.0 mm for the electrode fingers, and *w* = 4.0 mm for the gap between each WE finger.

The patterning of channels in PDMS was obtained through replica molding by mixing a silicone elastomer base solution with its curing agent (Sylgard 184) in a 10:1 ratio w/w. A total of nine microfluidic channels was created by adding this mixture onto a 3D-printed master mold (Formlab printer) and using adhesive tapes to delimit its sidewalls, followed by curing at 65 °C for 90 min. The PDMS piece was then pulled off from the mold and bonded to the MEC.

### 4.3 Chip Holder

A piece of poly­(lactic acid) (PLA) with dimensions of 100 mm × 47 mm was obtained by 3D printing using an extrusion printer of fused deposition modeling (3D Sethi3D AiPA3). This piece was designed with a 1 mm recess to accommodate standard glass wafers (75 mm × 35 mm) and includes an integrated sloped structure that guides discards toward a built-in reservoir.

### 4.4 Imaging

Images of the sensors were obtained by inverted light microscopy (Leica DMi8) and traditional optical microscopy (Zeiss West Germany axiophot), as indicated in the figure captions. To enable the imaging from the inverted microscope, the chips were inverted so that the PDMS substrate was facing downward. This step was required as the nontransparent Au electrodes would otherwise obstruct the image recording. Images of the PDMS microfluidic channels were also obtained by laser scanning confocal microscopy (Keyence VK-X200), whereas the nanostructured microelectrodes (NMEs) were characterized by scanning electron microscopy (SEM, FEI Quanta 650 FEG).

### 4.5 Flow Analyses

Fluid operations were performed with the aid of a syringe (NE-8000, New Era Pump Systems) or a peristaltic pump (Masterflex Ismatec 78001-82).

### 4.6 Adhesion Strength

We used a pressure controller (OB1MK4, Elveflow) and a 12-channel microfluidic switch (MUX Distribution 12, Elveflow) to regulate and route water flow through the microfluidic PDMS channels (3 mm × 200 μm) at pressures ranging from 0.5 to 2.0 bar. In addition, a Coriolis-based mass flow meter/controller (mini CORI-FLOW, Bronkhorst) was employed for precisely recording the ensuing flow rate. From flow rate vs pressure curve, we could measure the burst pressure by applying flow rates of up to 99.9 mL min^–1^ through a syringe pump (New Era Pump Systems Inc., NE-1000). To date, for the shelf life analysis, the devices were simply stored in vacuum plastic bags.

### 4.7 Electrochemical Analyses of the Redox Probe

The assays were made via a hand-held one-channel potentiostat (Sensit BT, Palm Sens). The supporting electrolyte consisted of a solution containing 1.0 mol L^–1^ KNO_3_ in 12.0 mmol L^–1^ PB. The SWV tests to [Fe­(CN)_6_]^3–/4–^ were conducted by applying a potential window ranging from −0.3 to +0.3 mV, a modulation amplitude of 35.0 mV, a step potential of 3.5 mV, and a frequency of 15 Hz. In these analyses, we used 800-μm WEs.

### 4.8 Electrodeposition of Nanostructured Microelectrodes

The NMEs were electrochemically grown (Metrohm Autolab potentiostat, PGSTAT 302N) on 45-μm recessed WEs utilizing the two-electrode on-MEC sensors. The electrodeposition occurred at room temperature in a solution containing 25.0 mmol L^–1^ HAuCl_4_ in HCl 1.0 mol L^–1^ employing distinct DC potentials (−0.2, −0.3, −0.5, −0.6, and −1.0 V vs Au) until the charge reached 120 μC. The solutions were added to PDMS channels utilizing a manual micropipette, whereas the electrodepositions were performed under a stationary condition.

### 4.9 Cell Proliferation and Electrochemical Monitoring

Cell proliferation was performed at 37 °C for 24 h under a CO_2_ atmosphere (Forma Series 3 Water Jacketed, Thermo Scientific) using DMEM supplemented with 10% FBS and 1% antibiotic solution (penicillin/streptomycin). In practice, suspensions of either MDA-MB-231 (10 × 10^4^ cells per PDMS channel) or HT-29 cells (15 × 10^4^ cells per PDMS channel) were added to PDMS channels with the aid of an automatic pipet (Xplorer plus, Eppendorf) under a controlled flow rate of 83 μL s^–1^. To electrochemically monitor the on-sensor proliferation of MDA-MB-231 and HT-29 cells, the same potentiostat mentioned above was used. SWV analyses of [Ru­(NH_3_)_6_]^3+^ at 6.0 mmol L^–1^ (electrolyte: DMEM with 10% FBS and 1% antibiotic solution) were made before and immediately after the cell proliferation step using 800-μm WEs. The SWV voltammograms were recorded from −0.6 to 0 V with 15 mV step, 35 mV amplitude, and 15 Hz frequency.

### 4.10 Drug Susceptibility Testing

Tests with 2D cancer cells (HT-29 line) were conducted to evaluate their response to distinct concentrations (5, 50, 100, and 200 μmol L^–1^) of the anticancer drug DOX. The assay consisted of two main stages performed in a CO_2_ incubator at 37 °C: (i) cell proliferation for 24 h and (ii) drug exposure for 4 h. DOX was also added to channels through the automatic pipet under the flow rate of 83 μL s^–1^. SWV measurements of 6.0 mmol L^–1^ Ru­(NH_3_)_6_^3+^ were conducted before and after DOX treatment. To quantify the true cell viability data from conventional method, the cells were washed 3× with PBS after drug incubation, and then Alamar blue (Invitrogen, 10% in DMEM) was added to each well. After a 3-h incubation at 37 °C, the supernatants were transferred to a clean 96-well plate and analyzed using a multimode microplate reader (Synergy H1, BioTek) with excitation and emission wavelengths set to 560 and 590 nm, respectively.

### 4.11 Phosphate Analyses

The phosphomolybdate complex was prepared according to the literature.^50^ Basically, a solution with 49.0 g L^–1^ sulfuric acid (H_2_SO_4_, Merck), 16.0% v/v acetone (C_3_H_6_O, Synth) and 3.7 g L^–1^ ammonium molybdate ((NH_4_)_6_Mo_7_O_24_.4H_2_O, Sigma-Aldrich), referred to as blank solution, was prepared to form the phosphomolybdate complex in the presence of phosphate. For measurements with the synthetic body fluids, a dilution of 100-fold was applied to all solutions. A hand-held potentiostat (PalmSens4) conducted SWV analyses from +0.2 V to −0.5 V vs Au with a step size of −10 mV, amplitude of 50 mV, and frequency of 40 Hz, with a preconditioning step of 1 s at +0.1 V.

### 4.12 Statistics

The sample size, *n*, has been quoted throughout the article. The LODs were calculated using the signal-to-noise ratio (S/N = 3).^53^ Moreover, the variations of the data (i.e., the error bars in graphics) consisted of confidence intervals for α = 0.05.

## Supplementary Material











## Data Availability

The data supporting the findings described in this study are available from the corresponding author upon reasonable request.

## Notes

Dropping solutions into the PDMS channels with the aid of a micropipette (MP4)
Rinsing solutions directly inside the PDMS channels with the aid of a micropipette (MP4)
Continuous operations with a syringe pump at high flow rate (MP4)
Serial SWV analyses to detect phosphate (MP4)
Complementary figures related to microfluidic channels, chips, dropping-, rinsing-, and pumping-based assays, chip holder to collect discards, adaptability of bonding, burst pressure tests to assess adhesion strength of bonding, morphology and analytical performance of NMEs, cell proliferation and drug susceptibility tests, and phosphate analysis, along with captions of videos showing analytical operations using the device and serial phosphate analyses (PDF)

## Abbreviations

MEC: meshed electrochemical chip
WE: working electrode
QRE: quasi-reference electrode
PDMS: polydimethylsiloxane
HMC: hand-bonded microfluidic chip
SWV: square wave voltammetry
CA: chronoamperometry
NME: nanostructured microelectrode
SEM: scanning electron microscopy
LSCM: laser scanning confocal microscopy
LOD: limit of detection
