# Correction to “Touch-Enabled Reversible Microfluidic Ultradense Chips for Convenient, High-Throughput Electrochemical Assays”

**DOI:** 10.1021/acsami.5c24327

**Published:** 2025-12-16

**Authors:** Pedro H. N. da Silva, Gabriela Zoia, Paula C. R. Corsato, Christian O. Silva, Gabriel J. C. Pimentel, Bruna M. Hryniewicz, Bruna Bragantin, Rodrigo S. Costa, Flávio M. Shimizu, Iris R. S. Ribeiro, Renato S. Lima

In the original published article, the burst pressure tolerated by the proposed reversible bonding approach was inadvertently reported as at least 5.1 MPa. Eventually, we noticed that this adhesion strength-related result was superestimated due to an experimental error in the burst pressure tests. As discussed next, the deployed touch-enabled method for reversibly bonding polydimethylsiloxane (PDMS) substrates (with outlets from their bottom) on ultradense multisensor SU-8-coated chips presented an adhesion strength on par with the burst pressures typically achieved by reversibly bonded microfluidic devices.^1^ Nevertheless, it is important to highlight that this correction does not impact the results or major conclusions of the article. To date, despite this similarity in adhesion strength, while the typical reversible bonding (based on PDMS substrates with outlets from their top side) incurred leakages, devices reversibly bonded to PDMS bearing on-bottom outlets could yield leakage-free 24-h proliferation of cancer cells at 37 °C and enhanced leakage resistance over device handling and fluidic operations. In this regard, future studies are required to get a comprehensive understanding of the reasons behind the enhanced leakage endurance of the latter method. One should also mention that the deployed reversible bonding offers additional advantages that were crucial to the method reported in the originally published article, i.e., power-free fluidic pumping utilizing simply a micropipette, lengthy shelf life, and reusability.

To attain the burst pressure into the PDMS microfluidic channels, pressure ramps were attained by varying the flow rate (Q). By fitting the slope of this pressure vs Q curve, we could obtain hydraulic resistance (Hr) that was then utilized to calculate the burst pressure (P) of the reversibly bonded chip/PDMS channel from eq 1:

1 $$\mathrm{Hr}=\frac{P}{Q}$$

While the prior equation was adopted in the original version of the article to determine P from the total experimental hydraulic pressure, we have just recently noticed that the total hydraulic resistance (Hr_system+device_) must be subtracted from the measured value without the device connected (Hr_system_) to guarantee the determination of accurate burst pressure values supported by the device itself. When it comes to analyses using microfluidic channels with the dimensions reported in the original article, i.e., 3.0, 1.0, and 25.0 mm in width (w), high (h), and length (L), respectively, it was observed that Hr_system_ and Hr_system+device_ were similar (∼47.3 Pa mL min^–1^; Figure 1A). From this outcome, the experimental setup was revealed to lack the sensitivity to detect the device-related hydraulic resistance, thereby implying the overestimationg of a burst pressure of at least 5.1 MPa (obtained for the flow rate of 99.9 mL min^–1^, the maximum value applied by the used syringe-pump, and that was endured by the bonded device).

**Figure 1:**
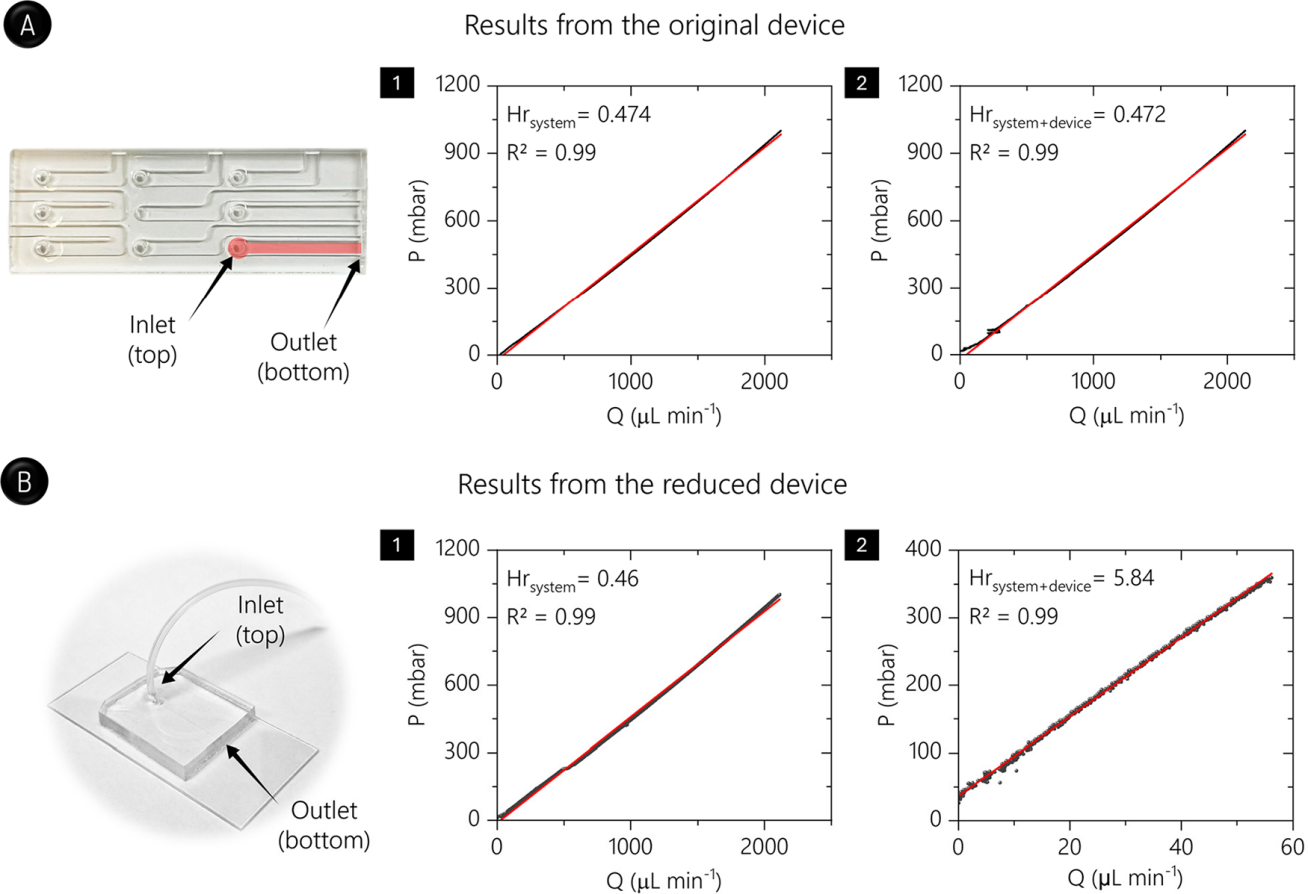
Burst pressure experiments. (A) Original microfluidic device, highlighting the channel under investigation and the corresponding pressure ramps for (1) the pumping system alone and (2) this system connected to the microfluidic device. (B) Microfluidic device with reduced channels (140.0 μm × 45.0 μm) and pressure ramps for (1) the pumping system alone and (2) this system coupled to the device. In both cases, PDMS was bonded against SU-8-coated glass, which served to simulate the ultradense chip.

In order to obtain the burst pressure experimentally, we evaluated a microfluidic channel with reduced dimensions of 140.0 μm (w), 45.0 μm (h), and 25.0 mm (L; Figure 1B). This new device was fabricated by photolithography,^1^ and the PDMS- SU-8-coated chip bonding procedure remained the same as that described in the original paper. Using this geometry, the instrumental setup demonstrated a sensitivity sufficient to detect the device-related hydraulic resistance. After subtracting Hr_system+device_ from Hr_system_, the Hr attributed solely to the microfluidic device was obtained as 0.5 kPa min μL^–1^, with the effective burst pressure of the hand-bonded microfluidic chips, as addressed in the original manuscript, being found to be 29.5 ± 1.2 kPa (*n* = 3; see Figure 1B)—the maximum flow rate tolerated by the device was 58.7 μL min^–1^. This adhesion strength is consistent with those of typical reversible PDMS bonding approaches, i.e., with substrates integrating outlets from their top side.^1^

The prior experimental burst pressure is consistent with the data theoretically predicted through transport phenomena equations, as described following. First, the average fluid velocity (v) inside the 140.0 μm × 45.0 μm channel at 58.7 μL min^–1^ is calculated through eq 2:^2^

2 $$v=\frac{Q}{A}=\frac{9.77\times 10^{-10}\frac{m^{3}}{s}}{\left(140\times 10^{-6}\times 45\times 10^{-6}\right)m^{2}}=0.15\frac{m}{s}$$

Because the channel is rectangular, the hydraulic diameter (*D*_h_) is obtained, as follows:

3 $$D_{h}=\frac{2\mathrm{wh}}{\left(w+h\right)}=\frac{2\times 140\times 45\times 10^{-12}m}{\left(140+45\right)\times 10^{-6}m}=0.610^{-4}m$$

The Reynolds number (Re) is next calculated, as shown below:

4 Re=ρv Dhμ=1000kgm3×0.15ms×0.610−4m10−3Pa s

which results in Re of 10.6. Since the flow is laminar (Re < 2300), as expected, eq 5 encompassing the previously attained parameters can be used to correlate P and v.

5 $$\frac{\Delta P}{Q}=f\frac{L}{{\mathrm{Q D}}_{h}}\rho \frac{v^{2}}{2}=R_{h}$$

Based on eq 6, the friction factor (f) is calculated as

6 f=72.04Re=6.8

In this way, by substituting all terms in eq 5, the theoretical burst pressure was calculated as 30.1 kPa, closely matching the experimental result of 29.5 kPa.

Having validated a theoretical route for calculating the burst pressure, we next sought to apply this strategy to estimate the pressure supported by the original 3.0 × 1.0 mm channel at a flow rate of 99.9 mL min^–1^ (the highest Q delivered by our syringe pump, and that was withstood by the reversibly bonded device, as aforesaid). This pressure was found to be 0.2 kPa. Finally, it is worthwhile highlighting that all authors agree with the described corrections and confirm that addressing such data issues does not impair the results or conclusions of the original article, as mentioned above. We sincerely apologize for the error and would like to reinforce our commitment to providing ACS readers with accurate, unbiased, and statistically reliable data.

## Experimental Section

We used a pressure controller (OB1MK4, Elveflow), a 12-channel microfluidic valve (MUX Distribution 12, Elveflow), and a mass flow meter (mini CORI-FLOW, Bronkhorst) to inject water at room temperature in the PDMS channels through pressure ramps, while precisely measuring the flow rates.
