# Supplementary material for: Touch-Enabled Reversible Microfluidic Ultradense Chips for Convenient, High-Throughput Electrochemical Assays
Source: ACS Appl Mater Interfaces. 2025 Jul 21;17(32):45847–58. doi: 10.1021/acsami.5c08760 (PMC12356537; doi:10.1021/acsami.5c08760)
Supplement: Supplementary file 5 [file am5c08760_si_005.pdf]

Supporting Information

Touch-Enabled Reversible Microfluidic Ultradense Chips for Convenient, High-Throughput Electrochemical Assays

Pedro H. N. da Silva,[†,‡] Paula C. R. Corsato,[†,‡] Christian O. Silva,[†,Ω] Gabriel J. C. Pimentel,[†,‡] Bruna M. Hryniewicz,[†] Bruna Bragantin,[†,‡] Rodrigo S. Costa,[Δ,†] Flávio M. Shimizu,[†] Iris R. S. Ribeiro,[†] and Renato S. Lima*[†,‡,⊥,‖]

[†]Brazilian Nanotechnology National Laboratory, Brazilian Center for Research in Energy and Materials, Campinas, São Paulo 13083-970, Brazil
[‡]Institute of Chemistry, University of Campinas, Campinas, São Paulo 13083-970, Brazil
[Ω]Department of Chemistry at Federal University of São Carlos, São Carlos, São Paulo 13565-905, Brazil
[⊥]São Carlos Institute of Chemistry, University of São Paulo, São Carlos, São Paulo 13565-590, Brazil
[Δ]Department of Chemistry, Federal University of Paraíba, João Pessoa, Paraíba 58051-900, Brazil
[‖]Center for Natural and Human Sciences, Federal University of ABC, Santo André, São Paulo 09210-580, Brazil
*Email of the corresponding author: renato.lima@lnnano.cnpem.br.

Contents:

1. **Complementary figures**
1.1. **Microfluidic channels**
1.2. **Meshed electrochemical chip**
1.3. **Dropping-based assays**
1.4. **Rinsing-based assays**
1.5. **Pumping-based assays and chip holder to collect discards**
1.6. **Adaptability of bonding**
1.7. **Burst pressure tests to assess adhesion strength of bonding**
1.8. **Calculation of the hydrostatic pressure**
1.9. **Morphology and analytical performance of NMEs**
1.10. **Cell proliferation and drug susceptibility tests**
1.11. **Phosphate analysis**
1.12. **Cost related to plasma usage**
2. **Movie captions.**

1. Complementary figures

1.1. Microfluidic channels

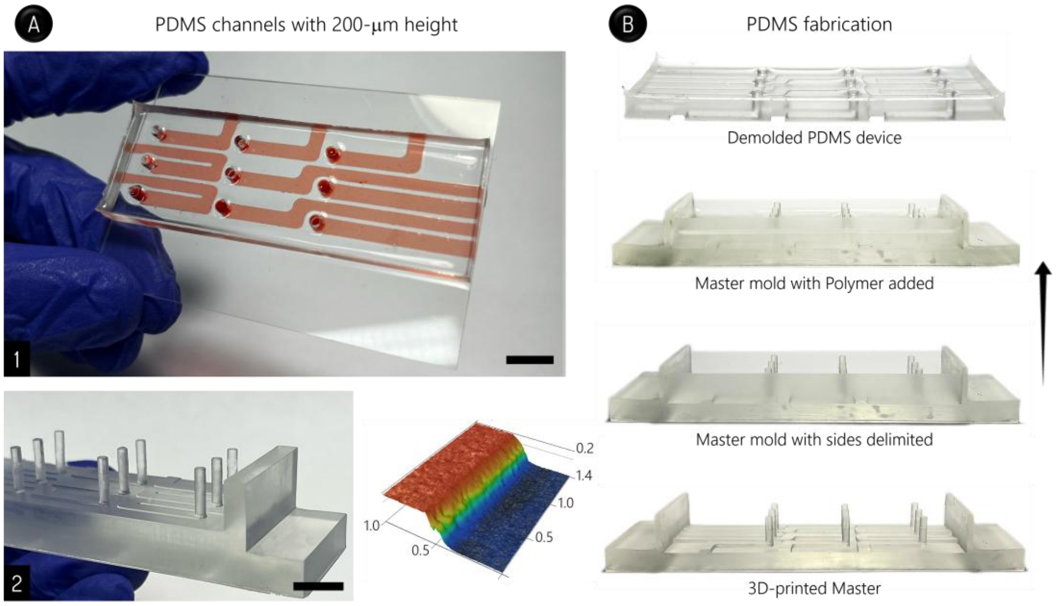

Figure S1. Touch-based reversible bonding. **(A)** Glass-against bonded substrate of polydimethylsiloxane (PDMS) with 3 mm × 200 µm channels (1) and 3D-printed master for molding the microfluidic channel patterns (2). Inset: laser scanning confocal microscopy (LSCM) image (scale in mm) of the channel walls. Scale bars: 1 cm. **(B)** Main steps of the replica molding approach to fabricate the channels through a 3D-printed master.

1.2. Meshed electrochemical chip

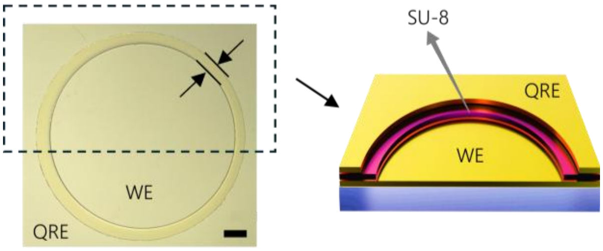

Figure S2. Electroactive area delimited by SU-8. The top quasi-reference electrode (QRE) is deposited on SU-8 at a certain retreat from the SU-8 edges that define the working electrode (WE) area, as highlighted by arrows in this optical image. Inset: illustration of the cross-section of a sensor. Scale bar: 100 µm.

1.3. Dropping-based assays

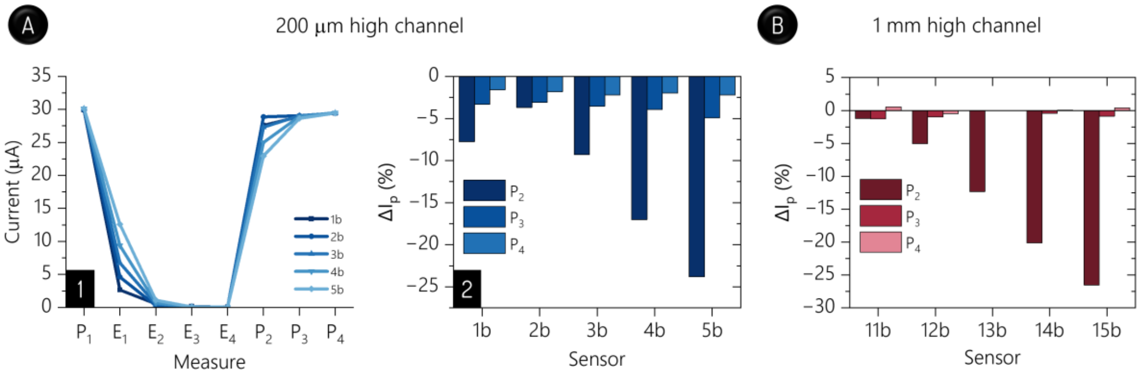

Figure S3. Square wave voltammetry (SWV) analyses of 5.0 mmol L^{-1} ferri/ferrocyanide ($[Fe(CN)_6]^{3-/4-}$) using meshed electrochemical chips (MECs) to assess the medium exchange in the PDMS channels via dropping-based tests using a manual micropipette. **(A)** SWV peaks (at −0.2 V) recorded by successively dropping 20 μL of electrolyte (E_i) and redox probe (P_i) utilizing a manual micropipette and a 3 mm × 200 μm channel (1), along with their relative standard deviations (Δi_p) when adding P_i (2). These deviations were calculated in relation to the control response (P_1). **(B)** Δi_p after successive additions of 60 μL P_i in a 3 mm × 1 mm channel. The complementary data using this channel are shown in **Figure 1D** (main text).

1.4. Rinsing-based assays

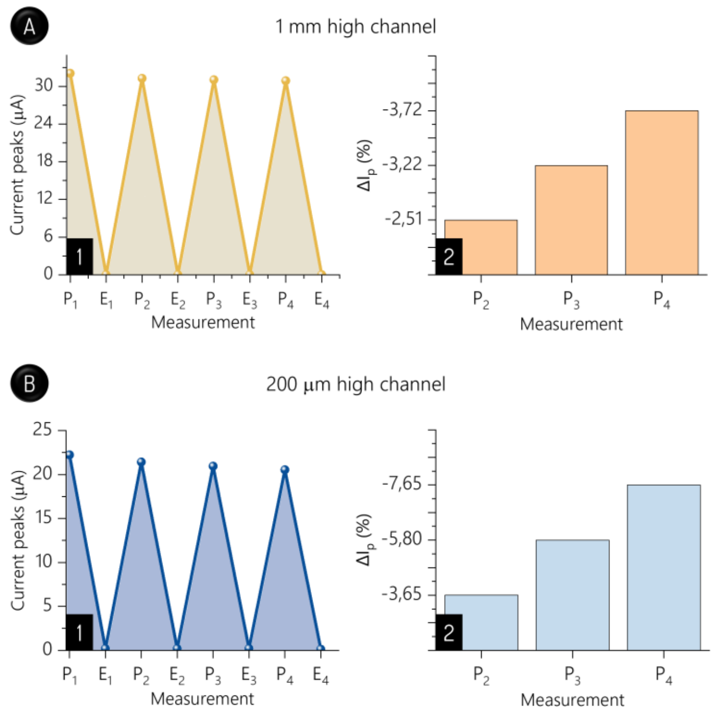

Figure S4. SWV analyses of 5.0 mmol L^{-1} $[Fe(CN)_6]^{3-/4-}$ to test the medium exchange in the PDMS channels via rinsing-based assays with a manual micropipette. **(A)** SWV peaks (at −0.2 V) recorded by successively rinsing 180 μL of E_i and P_i directly inside a 3 mm × 1 mm channel employing a manual micropipette (1), as well as their relative standard deviations, Δi_p, when adding P_i (2). **(B)** These same data of SWV (1) and Δi_p (2) for a 3 mm × 200 μm channel.

1.5. Pumping-based assays and chip holder to collect discards

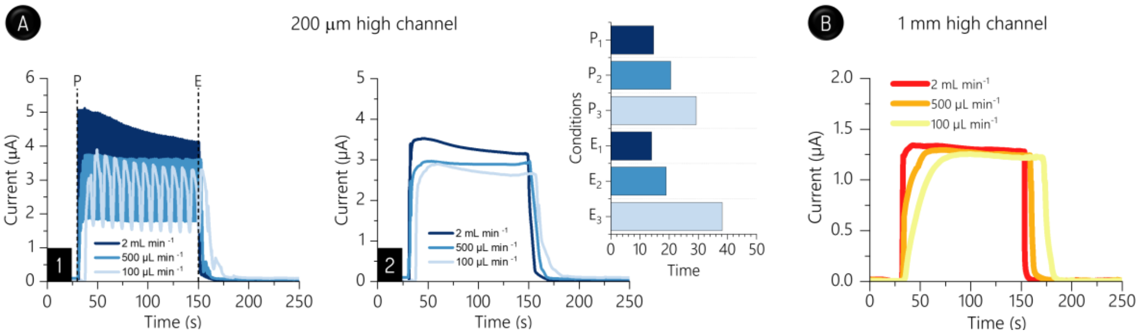

Figure S5. Chronoamperometry (CA) analyses of 10.0 mmol L^{-1} [Fe(CN)$_6$]$^{3-/4-}$ to evaluate the continuous operation of hand-bonded microfluidic chips (HMCs) using a peristaltic pump. **(A)** CA currents (at +0.5 V) monitored by alternatively flowing 10.0 mmol L^{-1} [Fe(CN)$_6$]$^{4-}$ and electrolyte inside a 3 mm × 200 µm channel at different flow rates, as indicated in the graphic (1). Plots with adjacent averaging for noise reduction (2), which were intended to find the time (in s) needed to reach stable currents for probe and electrolyte at 2.0 (P_1 and E_1), 0.5 (P_2 and E_2), and 0.1 mL min^{-1} (P_3 and E_3). These data are presented in the inset. **(B)** CA plots with adjacent averaging to reduce the noise of the data using a 3 mm × 1 mm channel. The complementary data using this channel are shown in **Figure 1E**.

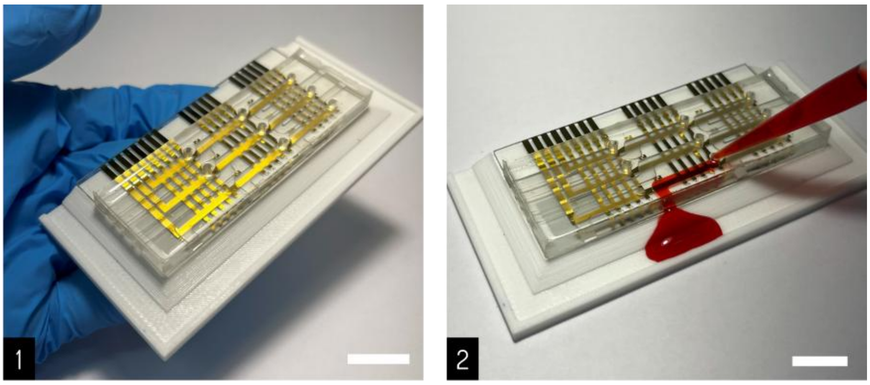

Figure S6. Chip holder. 3D-printed piece supporting the HMC without (1) and with discard (red) from fluid outlet at the bottom of the PDMS being collected by the holder (2). Scale bars: 1 cm.

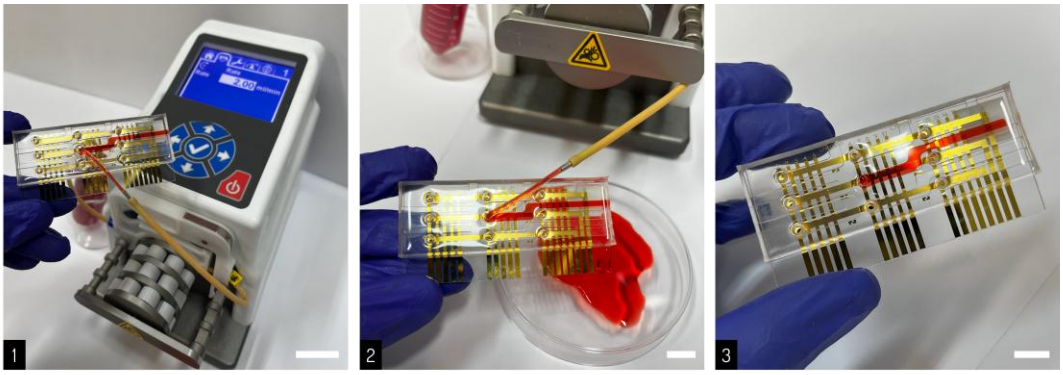

Figure S7. Peristaltic pump applying a flow rate of 2 ml min^{-1} inside a 3 mm × 1 mm channel (1). Device during (2) and after being interrogated with a constant flow rate (3). Scale bars: 2 cm (1) and 1 cm (2 and 3).

1.6. Adaptability of bonding

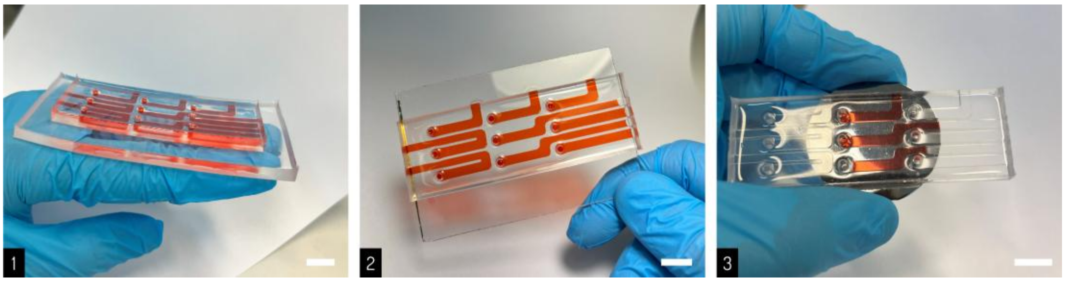

Figure S8. Microfluidic devices applied against PDMS (1), glass (2), and silica (3) after applying a flow rate of 2 mL min^{-1} for 5 min with the aid of a peristaltic pump. Scale bars: 1 cm.

1.7. Burst pressure tests to assess adhesion strength of bonding

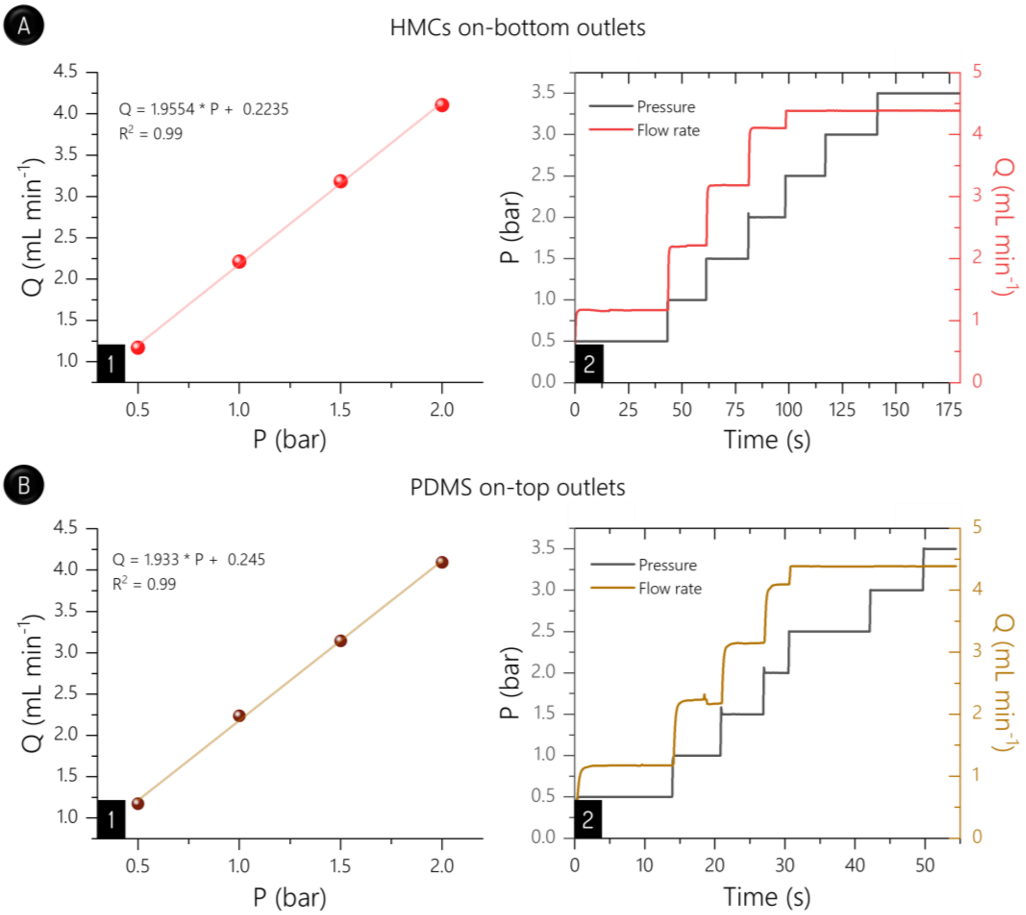

Figure S9. Tests of the adhesion strength of bonding utilizing 3 mm × 200 µm PDMS channels. **(A)** Linear relationship between pressure (P) and flow rate (Q) used to calibrate the system (1), along with the stepwise increase in P and corresponding Q for burst pressure tests used to evaluate adhesion strength of the HMCs (2). **(B)** Same prior data for the system with on-top outlets (1,2).

1.8. Calculation of the hydrostatic pressure

The pressure distribution in a static fluid within an L-shaped hydraulic circuit is shown in main text (diagram illustrated in **Figure 1A**). At the top point, located at the fluid surface, the fluid is subjected to the atmospheric pressure (P_0) in Campinas-SP (Brazil), 102,300 Pa. Since the fluid is at rest, the pressure at any point below the surface increases linearly with depth due to the weight of the fluid above it. At the lowest point, approximately 5 mm below the top point, the horizontal section of the pipe experiences higher pressure, which can be calculated using the hydrostatic pressure equation ($P = P_0 + \rho g h$). Substituting the values for water (ρ = 1,000 kg m^{-3}), gravitational acceleration (g = 9.81 m s^{-2}), and depth (h = 0.005 m), the pressure at the bottom point was calculated as 102,349.05 Pa (~0.1 MPa).

1.9. Morphology and analytical performance of NMEs

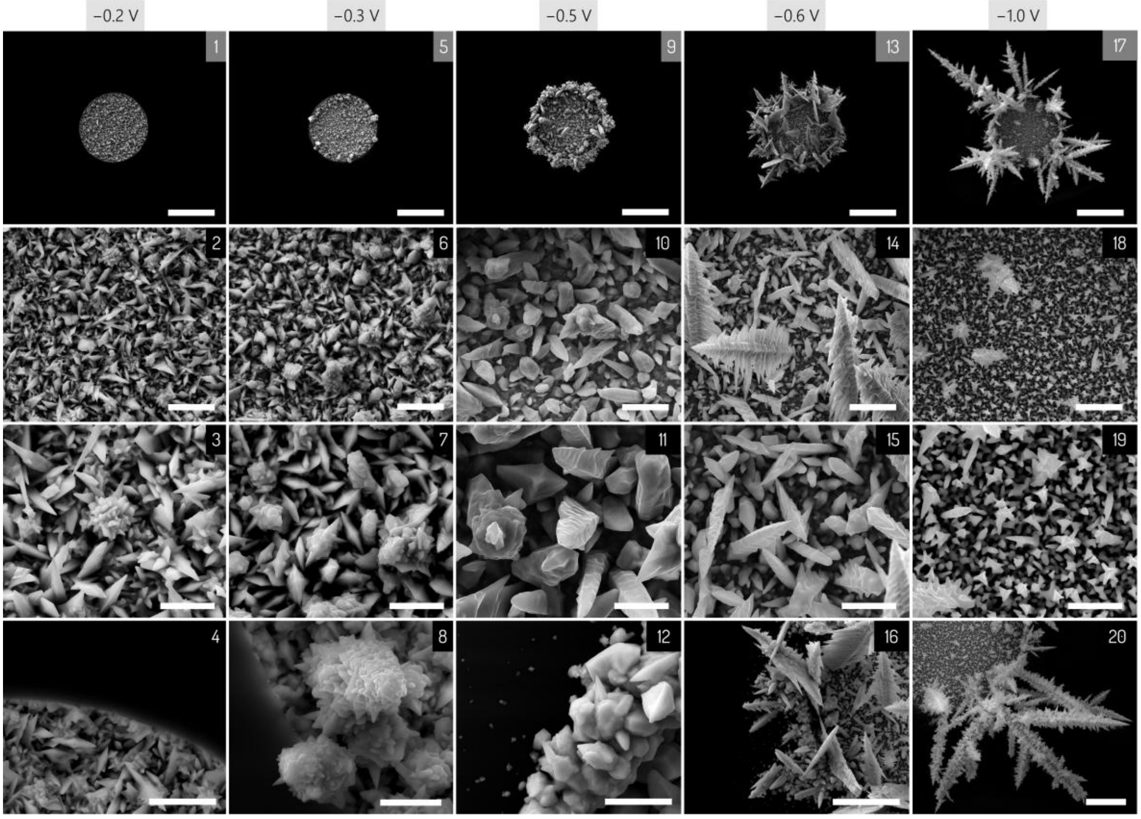

Figure S10. SEM images of the nanostructured microelectrodes (NMEs). Structures attained by applying distinct potentials, as stressed. Scale bars: 20 µm (1, 5, 9, 13, and 17), 10 µm (16 and 20), 4 µm (2, 6, 10, 14, and 18), 3 µm (4 and 12), and 2 µm (3, 7, 8, 11, 15, and 19).

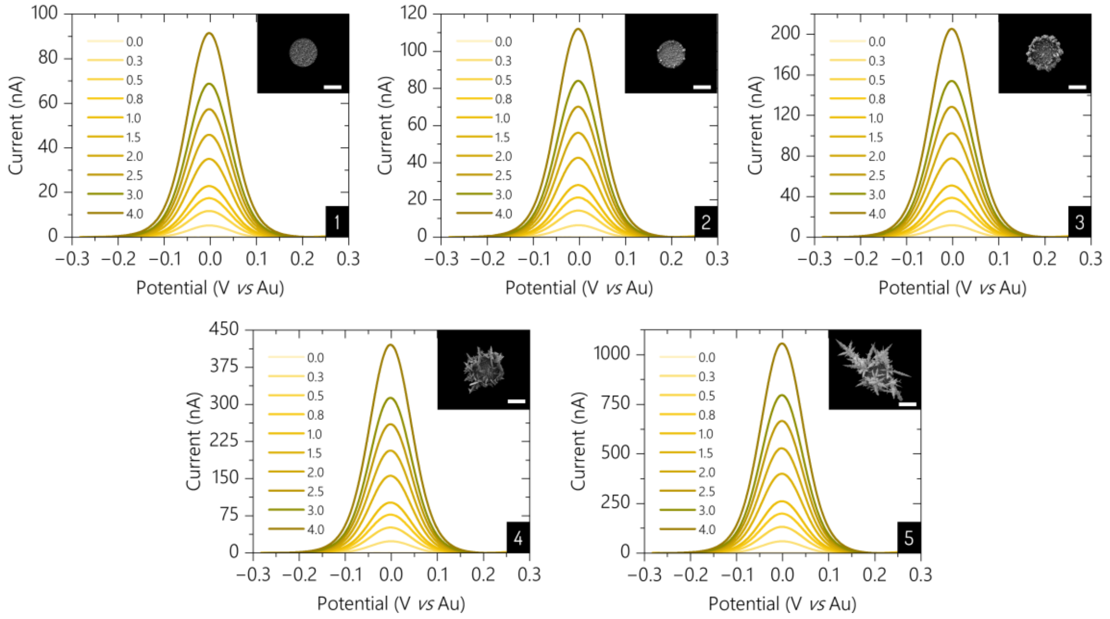

Figure S11. SWV scans for distinct concentrations (in mmol L^{-1}) of $[Fe(CN)_6]^{3-/4-}$ to assess the performance of the distinct NMEs. Structures obtained by applying −0.2 (1), −0.3 (2), −0.5 (3), −0.6 (4), and −1.0 V (5). Scale bars: 30 μm.

1.10. Cell proliferation and drug susceptibility tests

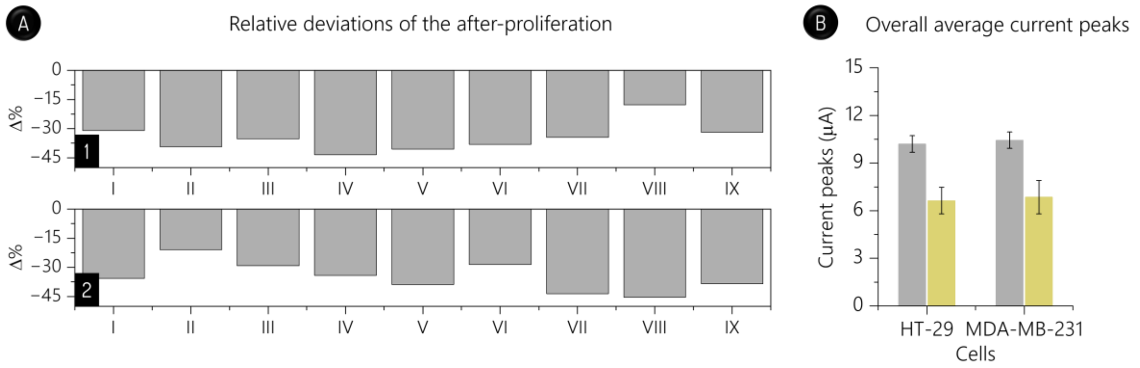

Figure S12. Cell proliferation. **(A)** Relative deviations (Δ%) of the after-proliferation SWV current peaks (in relation to the before-proliferation signals) for HT-29 (1) and MDA-MD-231 (2) cells. **(B)** Ensuing global average peaks. Cell adhesion SWV measurements (n = 15) were performed by monitoring 6.0 mmol L^{-1} hexaammineruthenium(II) ($[Ru(NH_3)_6]^{3+}$) before and after cellular proliferation.

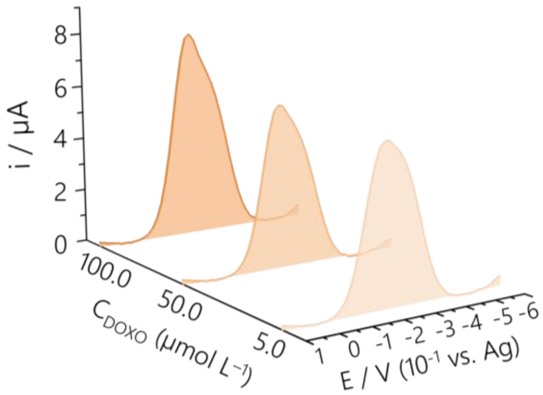

Figure S13. SWV responses to 6.0 mmol L⁻¹ $[Ru(NH_3)_6]^{3+}$ obtained after exposing HT-29 cells to increasing concentrations of DOX (C_{DOXO}), showing a progressive increase in current because of the DOX-induced cell detachment from the electrode surface.

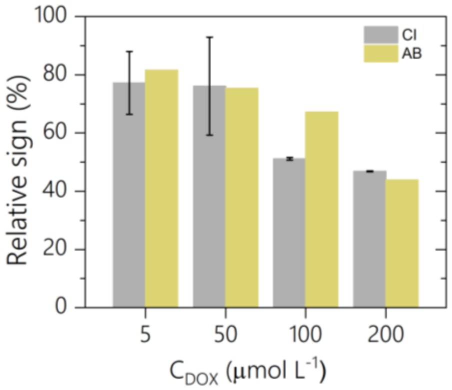

Figure S14. Drug susceptibility tests. Comparison between cell index (CI) and cell viability obtained by the Alamar blue (AB) method for HT-29 cells exposed to increasing DOX concentrations (5–200 µmol L⁻¹).

1.11. Phosphate analysis

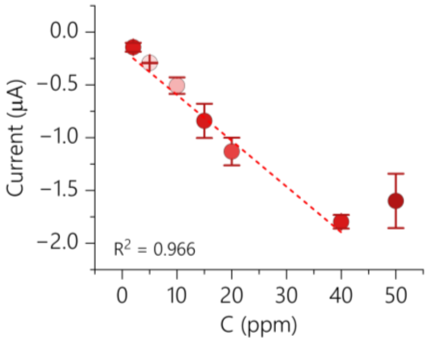

Figure S15. Analytical performance under stationary analyses of on-MEC droplets. Curve for phosphate standards at different concentrations.

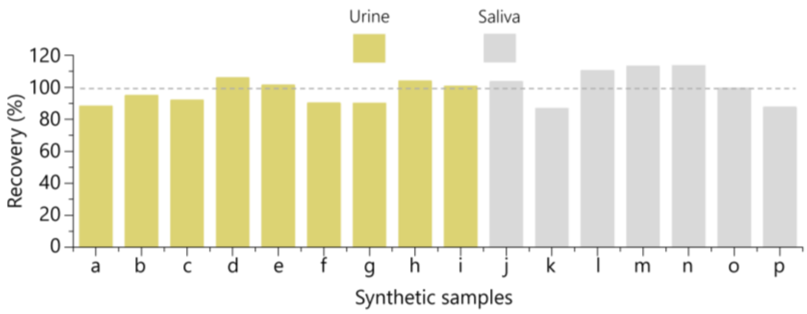

Figure S16. Recovery values obtained from electrochemical analyses of synthetic urine (a–i) and saliva (j–p) samples.

1.12. Cost related to plasma usage

The pricing calculation for the plasma treatment service relied on an hourly operational cost of approximately $10.00. This cost encompasses equipment usage, infrastructure, gas, and personnel salary. Assuming the operator can complete up to three processing cycles within one hour, treating a total of 24 chips per cycle (i.e., 72 chips per hour), the cost is found to be roughly $3.33 per batch.

2. Movie captions

Video S1. Dropping solutions into the PDMS channels with the aid of a micropipette.

Video S2. Rinsing solutions directly inside the PDMS channels with the aid of a micropipette.

Video S3. Continuous operations with a syringe pump at high flow rates.

Video S4. Serial SWV analyses to detect phosphate.
